# A multivariable analysis of the contribution of socioeconomic and environmental factors to blood culture *Escherichia coli* resistant to fluoroquinolones in high- and middle-income countries

**DOI:** 10.1186/s12889-022-12776-y

**Published:** 2022-02-19

**Authors:** Amy Booth, Astrid Louise Wester

**Affiliations:** 1 Faculty of Health Sciences, University of Cape Town, Rondebosch, Cape Town, 7700 South Africa; 2 Centre for Antimicrobial Resistance Research, and Division of Infection Control, Norwegian Institute of Public Health, Lovisenberggata 8, 0456 Oslo, Norway

**Keywords:** Antimicrobial resistance, Quinolone-resistant *Escherichia coli*, Wastewater, sanitation, and hygiene, Antimicrobial usage, Corruption

## Abstract

**Background:**

Antimicrobial resistance (AMR) is a public health concern. We wanted to determine if various environmental and socioeconomic variables as well as markers of antimicrobial use impacted on the level of AMR in countries of different income levels.

**Methods:**

We performed cross-national univariate and multivariable analyses using the national proportion of quinolone-resistant *Escherichia coli* (QREC) in blood culture as the dependent variable. Access to safe water and sanitation, other socioeconomic variables, and human and animal antimicrobial consumption were analysed.

**Results:**

In middle-income countries, unsafely managed sanitation, corruption and healthcare access and quality were significantly associated with the national proportion of blood culture QREC (%) in univariate analyses, whereas no variables remained significant in the multivariable models. For the multivariable high-income country model, corruption and healthcare access and quality were significantly associated with blood culture QREC (%) levels. For the model including all countries, human fluoroquinolone use, corruption level, livestock and crop production index were significantly associated with blood culture QREC (%) levels in the univariate analyses.

**Conclusion:**

Corruption is a strong predictor of AMR, likely reflecting a multitude of socioeconomic factors. Sanitation quality contributed to increased blood culture QREC (%) levels in middle-income countries, although was not an independent factor, highlighting the need to also focus on infrastructure such as sanitation services in the context of AMR.

**Supplementary Information:**

The online version contains supplementary material available at 10.1186/s12889-022-12776-y.

## Background

Antimicrobial Resistance (AMR), the ability of a disease-causing microbe to survive antimicrobial agents, is a growing global public health concern. The reduced options to treat infectious diseases can lead to longer duration of illness, increased hospital time and increased mortality rates across all areas of healthcare. AMR is currently estimated to account for 700,000 deaths annually worldwide, increasing to approximately 10 million deaths and US$ 100 trillion annually by 2050 [1].

The development and spread of AMR is a complex issue, partly due to the fact that environmental bacteria, predating the antibiotic era, carried genes that encode for resistance to antibiotics [2]. Indeed, antibiotics and antibiotic resistance genes are natural elements of the environment. The concern, however, is the influence that human activities, including the misuse of antibiotics and inadequate disposal of them, has had on driving the distribution and abundance of these resistance genes [2].

Antimicrobial agents are used in many societal sectors. Apart from their role in human and veterinary medicine, they are used extensively in agriculture, aquaculture and the farming industry. There is much emphasis on the impact of inappropriate antibiotic (here defined as antimicrobials with effect on bacteria only) use in human medicine on the development of AMR, resulting in the development of the World Health Organization (WHO) guideline on antibiotic stewardship [3], as well as in agriculture and farming, resulting in guidance on prudent use of antibiotics according to the World Organisation for Animal Health (OIE) intergovernmental standards [4]. While this is vital for the control of AMR, it neglects an alarming aspect of antibiotic usage – the disposal of these agents into the environment and the resulting potential breeding grounds for resistant bacteria through selective pressure.

The role of the environment and quality of sanitation and water infrastructure as drivers for the development and spread of AMR, in addition to rate of disease spread, has become a recent focus of research [5]. When environmental and pathogenic bacteria share a common habitat, novel antibiotic resistance genes develop through processes such as mutations, rearrangements and horizontal gene transfer [5]. Stressors in the environment, primarily antimicrobials, but also metals and biocides, add to this process and induce the transfer of genetic material between organisms [6]. Different antimicrobials are also affected to varying extent by the environmental matrix (air, soil or water) that they enter. Some antimicrobials such as fluoroquinolones and sulphonamides, are chemically stable and are frequently detected in the environment, whereas others, like beta-lactams are easily degradable and not typically detected, although the wide use of these family of antibiotics can still contribute to resistance [7]. The temperature of the environmental matrix also influences the transfer of resistance genes with optimum transfer occurring at temperatures of thirty degrees Celsius (30 °C) [7].

The correlation between socioeconomic factors and AMR has also become a focus of research. Alvarez-Uria *et. al.* showed that a country’s income predicts their levels of AMR, with an 11·3% decrease in the prevalence of *E. coli* resistant to broad-spectrum cephalosporins for each log increase in Gross National Income (GNI) per capita [8]^.^ This study did not consider any potential confounders. The authors’ proposed explanation was that countries with a lower income have a higher population density, suboptimal sewage systems, poor sanitation and lack of access to clean water as well as inadequate antimicrobial stewardship programmes, low vaccination rates and lower education levels [8]. Collignon *et.al.* looked at the contribution of poor governance and corruption in European countries on AMR levels and demonstrated that higher levels of corruption are strongly associated with an increase in AMR [9]. They proposed that when the quality of governance is low, there are less effective antibiotic stewardship programmes and inadequate control of food and water safety. They also recently published a similar article, based on data from a broader geographical area, that found that poor governance, income level and lack of infrastructure contribute to AMR [10]. These studies highlight the importance of considering systems level factors in combination with data on antibiotic use, sanitation, water treatment, and other environmental factors, in investigating determinants of AMR. Indeed, Noyes *et.al.,* published a paper that emphasised the need to move away from solely focusing on antibiotic use and AMR, and to consider anthropogenic and environmental drivers of AMR [11].

Some of the most robust data on water and sanitation is produced by the World Health Organization (WHO) and United Nations Children’s Fund (UNICEF) Joint Monitoring Programme (JMP) for Water Supply, Sanitation and Hygiene (WASH) [12]. The JMP biannual report presents the proportion of the population with access to an improved sanitation facility and an improved drinking water source.

In order to further evaluate the impact of water and sanitation on AMR, along with various socioeconomic, environmental and agricultural factors, we performed a cross-national multivariable analysis. Furthermore, we wanted to assess whether the determinants of AMR remain consistent across high-income, middle-income and low-income countries. For example, while poor infrastructure may drive AMR in low-income countries, other factors may be at play in high-income countries where the standard of infrastructure remains consistently high. An answer to this could promote a more stratified response to the issue of AMR according to the drivers of AMR specific to each country.

## Methods

### Data acquisition

We chose to use the proportion of quinolone-resistant *Escherichia coli* (QREC %) in blood culture samples as our model pathogen and dependent variable. This was chosen as an indicator for national levels of AMR as *E. coli* is the prototypical faecal indicator bacterium [13]. Furthermore, *E. coli* is the target bacterial species of the WHO Tricycle AMR Surveillance Project, which focuses on the proportion of extended-spectrum beta-lactamase (ESBL) producing *E. coli* across the human, animal/food safety and environment compartments [13]. Fluoroquinolones are antibiotics that are stable in the environment and have widespread use in human and animal medicine [14]. They are in the ‘highest priority’ WHO list of critically important antimicrobials based on their essential use in human medicine and their high resistance potential [15].

Data on blood culture QREC (%) is available across multiple countries. We obtained this data from the Resistance Map Repository, produced by the Centre for Disease Dynamics, Economics and Policy (CDDEP). This database has aggregated data from 1999 to 2017 on antibiotic resistance from several sources including national and regional AMR surveillance databases [16]. CDDEP has harmonised the data to present similar definitions of resistance across countries to enable comparison. Each country and region included in the CDDEP database used standardised methods of testing for resistance, either using Clinical Laboratory Standards Institute (CLSI) criteria or European Committee on Antimicrobial Susceptibility Testing (EUCAST) breakpoints. This database presents data for a pathogen only when 30 or more isolates had been tested against an antibiotic agent. Fluoroquinolones included were ciprofloxacin, ofloxacin, levofloxacin, moxifloxacin and norfloxacin. The Resistance Map dataset was supplemented with data from the WHO Global Antimicrobial Resistance Surveillance System (GLASS) [17]. For countries that had data on proportion of QREC (%) among blood culture *E. coli,* we retrieved data on potential explanatory variables (Table 1).

**Table 1:** Description and sources of variables used

	Definition	Unit	Data Source (data year)
**Fluoroquinolone Resistant** ***E. coli***	Proportion of blood culture *E. coli* isolates tested per country that were resistant to at least one fluoroquinolone (ciprofloxacin, ofloxacin, levofloxacin, moxifloxacin, norfloxacin)	%	CDDEP Resistance Map (2020) [16] WHO GLASS Report Early Implementation (2017–2018) [17]
**Unsafely Managed Sanitation**	Proportion of a country’s population using a sanitation service that does not meet the criteria for safely managed sanitation, that is “use of improved facilities that are not shared with other households and where excreta are safely disposed of in situ or transported and treated offsite”	%	WHO/UNICEF JMP Report on Progress on Drinking Water, Sanitation and Hygiene (2017) [12]
**Unsafely Managed Water**	Proportion of a country’s population using a drinking water service that does not meet the criteria for safely managed drinking water, that is “drinking water from an improved water source that is located on premises, available when needed and free from faecal and priority chemical contamination”	%	WHO/UNICEF Joint Monitoring Program Report on Progress on Drinking Water, Sanitation and Hygiene (2017) [12]
**Human Fluoroquinolone Consumption**	Defined Daily Doses (DDD) of fluoroquinolones (ciprofloxacin, ofloxacin, levofloxacin, moxifloxacin, norfloxacin) consumed per 1000 population per year	DDD/1000/y	CDDEP Resistance Map (2015) [8, 16]
**Total Human Antibiotic Consumption**	DDD of all antibiotics consumed per 1000 population per year	DDD/1000/y	CDDEP Resistance Map (2015) [16]
**Animal Antimicrobial Consumption**	Total antimicrobial consumption by animals per year in mg per population correction unit (PCU)	Mg/PCU	CDDEP Resistance Map (2013) [16]
**Population Density**	Population per square kilometre of land area	Pop/km^2^	World Bank (2019) [18]
**Gross National Income**	The sum of value (in US dollars) added by all resident producers in a country plus any product taxes not included in the valuation of output plus net receipts of primary income from abroad, divided by the mid-year population	US dollars	World Bank (2019) [19]
**Corruption Perceptions Index**	A ranking of countries, on a scale of 1 to 100, based on their perceived levels of corruption (misuse of public power for private benefit), as determined by expert assessments and opinion surveys	NA	Transparency International (2019) [20]
**Education Level**	The mean number of completed years of education	Years	Our World in Data – Global Education (2017) [21]
**Healthcare Access and Quality Index**	A score out of a total of 100 based on measuring mortality rates from diseases that should not be fatal in the presence of effective medical care	NA	Our World in Data -Healthcare Access and Quality (2015) [22]
**Average Annual Temperature (degrees Celsius)**	Average national temperature in degrees Celsius	°C	Lebanese Economy Forum (2015) [23]
**Livestock Production Index**	Index of production of meat and milk from all sources, dairy products such as cheese, and eggs, honey, raw silk, wool and hides and skins relative to the base period 2004–2006	NA	World Bank (2016) [24]
**Crop Production Index**	Index of agricultural production for each year relative to the base period 2004–2006	NA	World Bank (2016) [25]
**Aquaculture Production**	Quantity of cultivated fish and crustaceans taken from marine and inland waters and sea tanks in metric tons	Metric Tons	World Bank (2016) [26]

*CDDEP* Center for Disease Dynamics Economics and Policy, *WHO* World Health Organization, *NA* Not applicable, *UNICEF* UN Children’s Fund, *JMP* Joint Monitoring Program, *WIDE* World Inequality Database on Education, *UNESCO* UN Educational, Scientific and Cultural Organization

### Statistical analysis

Countries included in the study were categorized into high-income, middle-income and low-income groups according to the updated World Bank classification [27]. There was only one low-income country with data on blood culture QREC (%), namely, Malawi.

Descriptive statistics were obtained for all variables using medians with interquartile ranges (IQR’s). Given all variables were numerical and fairly normally distributed we used univariate linear regression analysis to determine the association between individual independent variables and the outcome variable (proportion of QREC (%) among blood culture *E. coli* isolates tested). Regression coefficients and *p*-values for the independent variables analysed using univariate regression can be found in the Additional file 1. We developed scatter plots with correlation coefficients (R^2^) to describe the direction and strength of associations between the dependent variable and potential explanatory variables.

We performed multivariable linear regression to determine the effect of each variable when controlling for the others. All variables that were significantly associated with the outcome variable (*p*-value < 0·05) in the univariate analyses were included in a multivariable linear regression model. We performed analyses for and reported on separate multivariable models for all countries, high-income countries and middle-income countries. We also attempted to further stratify countries by income by testing models looking at upper-middle and lower-middle income countries, but we found that there was too little data in these sets and were concerned about low statistical power. We considered removing Malawi from the data-set as it was the only lower-income country, however, removing it made no impact on the significance of the results and we decided to analyse it in the middle-income country group for completeness of the data set. A *p*-value of less than 0·05 was considered significant in the multivariable regression models. The fit of each model was described using the coefficient of determination, R^2^.

Prior to inclusion in the multivariable models, we checked variables for heteroscedasticity and multicollinearity. Robust standard errors were used to account for heteroscedasticity. Variance Influence Factor (VIF) was used to test for multicollinearity, with a VIF of > 6 considered as potentially serious multicollinearity. We used the Missing Completely at Random (MCAR) for multivariate data test to detect any bias in the dataset.

STATA version 14.0 (Statacorp LLC, Texas, USA) was used for statistical analysis. Microsoft Excel 2019 was used to illustrate the proportion of QREC (%) among blood culture *E. coli* by country.

## Results

**Fig. 1:**
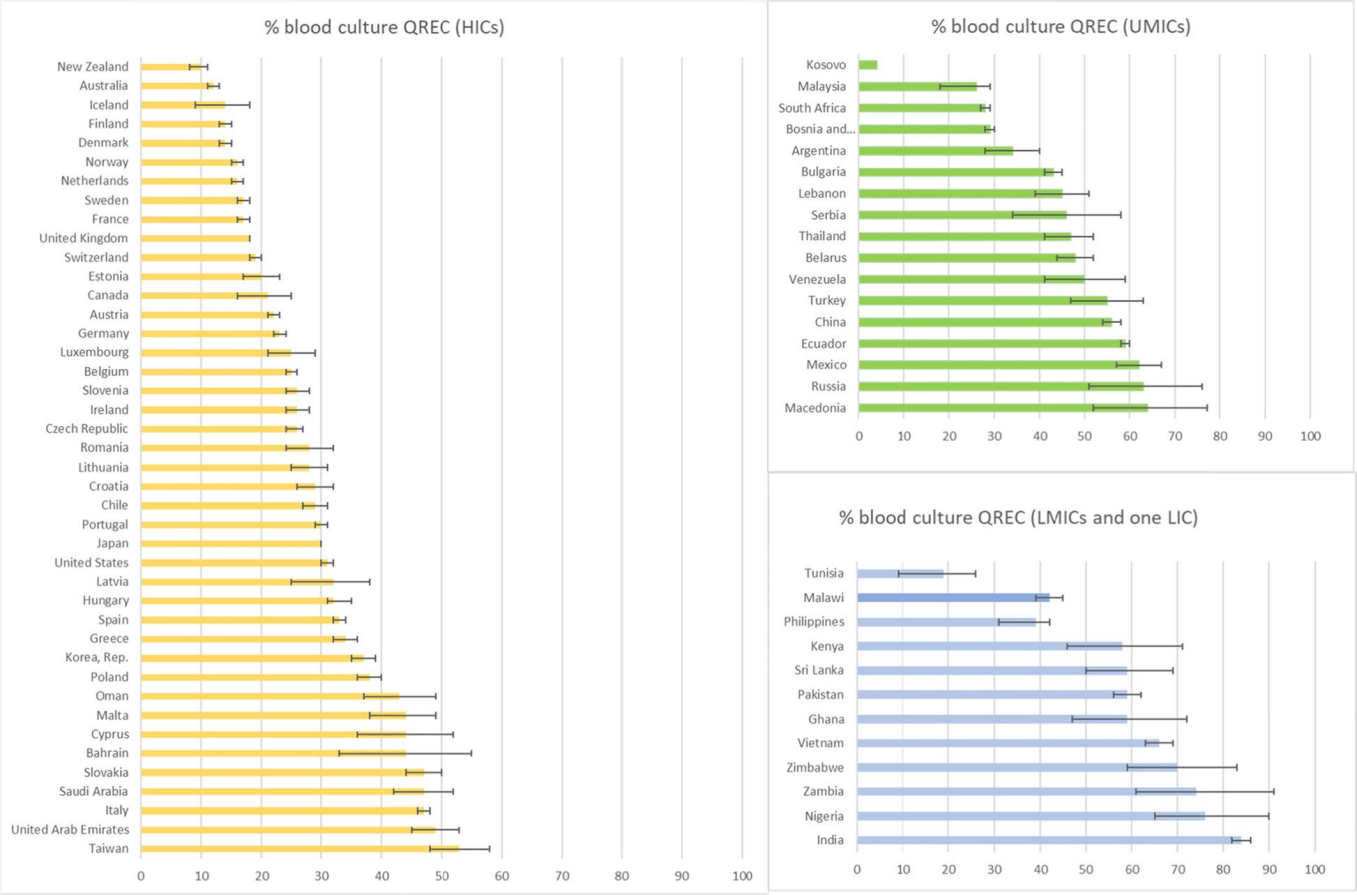
Proportion of QREC (%) among blood culture isolates of *E. coli*, by country and income level. Data on QREC (%) among blood culture *E. coli* were extracted from the Center for Disease Dynamics, Economics and Policy (CDDEP) Resistance map, for which sources were the World Health Organization’s Global Antimicrobial Surveillance System (GLASS), regional databases such as European Antimicrobial Resistance Surveillance Network (EARS-net), Central Asian and Eastern European Surveillance of Antimicrobial Resistance Network (CAESAR), and country-based or single institution-based systems. For most countries, the year of data production was 2016 or 2017, however some were produced in 2015 (New Zealand, Mexico, Zimbabwe, Kenya), 2014 (Canada, Chile, Ecuador, Kosovo), 2013 (Venezuela), and 2009 (Sri Lanka)

Seventy-one countries had data on blood culture QREC (%), of which 42 represented high-income countries, 28 were middle-income countries, and one country (Malawi) was a low-income country (Fig. 1). The median proportion of blood culture QREC (%) globally was 34%. Table 2 shows the number of countries that had data on dependent and independent variables as well as the median and interquartile range for each variable.

**Table 2:** Descriptive statistics

		Number of countries with data on potential explanatory variables	Median level (IQR)
**Dependent Variable**	Blood culture QREC (%)	71	34 (25–49)
**Water and Sanitation**	Unsafely Managed Sanitation (%)	67	2 (0–9)
	Unsafely Managed Water (%)	66	0 (0–5·3)
**Antibiotic Consumption**	Human Fluoroquinolone Consumption (DDD/ 1000 population/ year)	57	722 (426–1129·5)
	Total Human Antimicrobial Consumption (DDD/ 1000 population/ year)	57	7760 (5868–11,030·5)
	Animal Antimicrobial Consumption (mg/PCU)	54	60 (38–77)
**Socioeconomic Indexes**	Population Density (population per square kilometre)	69	93 (34–198·5)
	Gross National Income (US dollars)	70	16,935 (6248–42,467·5)
	Corruption Perceptions Index (score 1–100, higher score indicative of lower corruption levels)	70	53 (39–73·2)
	Education Level (years)	68	11 (9–12)
	Healthcare Access and Quality (score 1–100)	70	79 (71–87)
**Agricultural Indexes**	Average Annual Temperature (degrees Celsius)	67	11 (8–22)
	Livestock Production Index	69	109 (97–129·5)
	Crop Production Index	69	110 (95–128·5)
	Aquaculture Production Index	68	30,630 (6687–216,186·5)

**Fig. 2:**
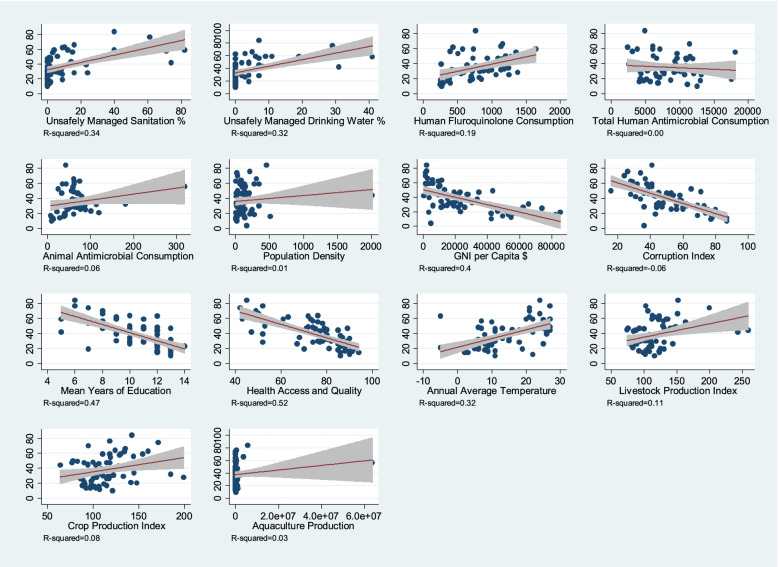
Scatterplot graphs of the linear regression analysis for all variables in all countries. Strengths of the associations are given in R-squared values

As visualised in Fig. 2 and calculated in the univariate analysis (see Additional file 1) several variables were positively correlated with an increased level of blood culture QREC (%) at *p* < 0·05, namely, unsafely managed sanitation, unsafely managed water, human fluoroquinolone consumption, average annual temperature, livestock production index (quantity of production of meat and milk relative to base period 2004–2006) and crop production index (quantity of agricultural production relative to base period 2004–2006). An increase in GNI per capita, corruption perceptions index (a higher score in the index is indicative of lower corruption levels), education level and healthcare access and quality were negatively correlated with the level of blood culture QREC (%).

For the univariate linear regression models including only high-income countries human fluoroquinolone consumption, animal antimicrobial consumption, population density, and average annual temperature were positively associated with an increase in blood culture QREC (%) at *p* < 0·05. GNI per capita, corruption perception index, education level, healthcare access and quality were negatively associated with an increase in blood culture QREC (%) at *p* < 0·05.

For the univariate linear regression models including only middle-income countries and one low-income country, an increase in unsafely managed sanitation was significantly associated with an increase in blood culture QREC (%), while an increase in corruption perception index and healthcare access and quality were significantly associated with a decrease in blood culture QREC (%).

In the multivariable linear regression model with all countries that had complete data (*n* = 53, Table 3*, All Countries column*), the model explained 73% of the total variation in blood culture QREC (%). Human fluroquinolone consumption and corruption perception index remained significant at *p* < 0·01. A one-unit improvement in the corruption perception index score resulted in a decrease of blood culture QREC (%) isolates by 0·45% if all other variables remain unchanged, while a one-unit increase in human fluroquinolone consumption resulted in a 0·01% increase in blood culture QREC (%) isolates. Livestock production index and crop production index also remained significant in this model with an increase in production relative to the baseline period being positively associated with blood culture QREC (%).

**Table 3:** Multivariable linear regression model results for independent risk factors for blood culture QREC (%) isolates tested

	Blood Culture QREC (%) Regression Coefficient (Robust Standard Error)		
	All Countries	High-Income Countries	Middle-Income Countries §
**Unsafely Managed Sanitation (%)**	0.09 (0.39)	–	0.13 (0.16)
**Unsafely Managed Water (%)**	0.39 (0.44)	–	–
**Human Fluoroquinolone Consumption (DDD/ 1000 population/ year)**	0.01 (0.00) ***	0.01 (0.01)	–
**Gross National Income (US dollars)**	0.00 (0.00)	0.00 (0.00)	–
**Corruption Perceptions Index (score 1–100)**	−0.45 ((0.16) ***	−0.29 (0.14) **	− 0.58 (0.38)
**Education Level (years)**	−0.22 (1.45)	− 0.61 (1.09)	–
**Healthcare Access and Quality (score 1–100)**	− 0.03 (0.31)	− 0.64 (0.29) **	− 0.23 (0.41)
**Average Annual Temperature (degrees Celsius)**	0.29 (0.26)	0.242 (0.27)	–
**Livestock Production Index**	0.17 (0.08) **	–	–
**Crop Production Index**	0.16 (0.07) **	–	–
**Animal Antimicrobial Consumption (mg/PCU)**	–	0.02 (0.04)	–
**Population Density (population per square kilometre)**	–	0.01 (0.01)	–
**Constant**	10.90 (38.44)	93.96 (33.98) **	83.63 (26.69) ***
**Observations**	53	33	26
**R-squared**	0.73	0.73	0.29

*DDD* Defined Daily Doses

*§Malawi included*

*** *p* < 0.01, ** *p* < 0.05, * *p* < 0.1

In the multivariable linear regression model with only high-income countries (*n* = 33; Table 3*, High-Income Countries column*), corruption perception index and healthcare access and quality remained negatively and positively associated with blood culture QREC (%) at *p* < 0·05 respectively, with this model also explaining 73% of the total variation in blood culture QREC (%). In the model with only middle-income countries Table 3, *Middle-Income Countries column*), there were only 26 observations and no variables remained significantly associated with blood culture QREC (%).

As shown in Table 4, animal antimicrobial consumption had the most missing data (17 missing values). When using Little’s MCAR (Missing Completely at Random) Test, only human fluoroquinolone consumption, total human antimicrobial consumption and animal antimicrobial consumption had *p* values < 0·05 indicating that the values of these variables are not missing at random. The remainder of the variables were missing completely at random.

**Table 4:** Tests for bias: Number of missing observations; Little’s MCAR Test; Chi-square Test

	Number of missing observations	MCAR’s Test: Prob > chi-square
**Unsafely Managed Sanitation (%)**	4	0·90
**Unsafely Managed Water (%)**	5	0·85
**Human Fluoroquinolone Consumption (DDD/ 1000 population/ year)**	14	0·01
**Total Human Antimicrobial Consumption (DDD/ 1000 population/ year)**	14	0·01
**Animal Antimicrobial Consumption (mg/PCU)**	17	0·03
**Population Density (population per square kilometre)**	2	0·41
**Gross National Income (US dollars)**	1	0·39
**Corruption Perceptions Index (score 1–100)**	1	0·14
**Education Level (years)**	3	0·36
**Healthcare Access and Quality (score 1–100)**	1	0·06
**Average Annual Temperature (degrees Celsius)**	4	0·83
**Livestock Production Index**	2	0·47
**Crop Production Index**	2	0·47
**Aquaculture Production Index (metric tons)**	3	0·31

The *p* value of the Shapiro-Wilk test for normality of data for our dependent variable (HO = blood culture QREC % is normally distributed) was *p* = 0.10 indicating that we cannot reject that the data was normally distributed. The mean Variance Inflation Factor was 4·10 which is less than 6·00, the indicator for multicollinearity. Finally, the *p* value of the Breusch-Pagan Test for heteroscedasticity for all the models was *p* < 0·01 (HO = error variances are all equal) indicating that we can reject the hypothesis that the data is homoscedastic and assume that the variability of the variables is unequal. Heteroscedasticity was adjusted for by using robust standard errors.

## Discussion

The potential health impact from AMR has prompted researchers to determine the main drivers for the development and spread of AMR. Several studies have assessed the effect of socioeconomic factors, including income level, corruption and infrastructure, on promoting AMR [8–10]. Our study adds to this body of research, while also drawing on water and sanitation quality data from the WHO/UNICEF JMP to investigate how these factors contribute to AMR, both irrespective of country income as well as after stratification according to income level. Our study also supports the links between AMR and water and sanitation issues, as underlined in the recent WHO/FAO/OIE technical brief on water, sanitation, hygiene and waste management to prevent infections and reduce the spread of AMR [28].

An increase in unsafely managed drinking water was significantly associated with an increase in blood culture QREC (%) when analysed with univariate regression in the combined model and the middle-income model, although it lost its significance in the multivariable models. We suggest that there was too little variance in the data to draw the conclusion that unsafely managed drinking water contributed independently to blood culture QREC (%) levels.

In the univariate analysis, we found that as the proportion of unsafely managed sanitation increased, so too did the levels of blood culture QREC (%). Poorer quality sanitation results in an environmental breeding ground for antibiotic resistance and thus explains the positive relationship with blood culture QREC (%) [29]. Increased unsafely managed sanitation was significantly associated with an increase in blood culture QREC (%) in the univariate model including only middle-income countries, but not the model including only high-income countries. One can infer that there is a fairly similar and high level of sanitation quality in high-income countries, whereas in middle-income countries the variance is greater, and it shows its significance. This finding agrees with the recently published study by Collignon *et. al.* which found a positive correlation between infrastructure (i.e. access to sanitation), and AMR levels [10]. In the multivariable models, unsafely managed sanitation loses its significant association with blood culture QREC (%), possibly because there are too few data points among lower-income countries where one expects that sanitation may play a larger role in AMR.

Human fluoroquinolone consumption had a positive association with blood culture QREC (%) level in the multivariable model including all countries, and when analysed in high-income countries only, but not for middle-income countries. A vast body of research shows that high consumption of antimicrobials is a main driver of AMR. Most of these studies, however, are performed in high-income settings, and have not included possible socioeconomic and environmental factors. Collignon *et. al.* on the other hand did include these additional factors and found that antibiotic use was not significant in any of the multivariable models [10]. Their findings, and ours, suggest that, especially in middle-income countries, and possibly also in low-income countries, other variables are also at play.

An increase in GNI per capita was significantly associated with an increase in blood culture QREC (%) in the univariate analyses in both the combined and high-income countries only analyses, however, it lost its significance in the multivariable models. This positive association is consistent with the findings of Collignon *et. al.* who explained it by the fact that wealthier countries use more antibiotics [10]. The result differs, however, from the study performed by Alvarez-Uria *et. al.* where they found that income was inversely related to AMR, although no other variables were considered in this study [8].

The corruption perceptions index (the higher the score in the index, the lower the corruption) remained strongly negatively associated with blood culture QREC (%) across all models, being the only significant variable (*p* value < 0·05) in all three univariate analyses. It also remained significant in the multivariable models that analysed all countries and in the high-income countries only, although it lost its significance in the middle-income multivariable model, likely due to too few observations. It is probable that countries with more corruption will have fewer antibiotic stewardship programmes and less stringent policies on the disposal of pharmaceuticals, as well as worse infrastructure and water and sanitation services.

Previous research indicates that the transfer of resistant genes occurs at an optimum temperature of 30 °C meaning that countries with higher average temperatures are more likely to produce optimal conditions for bacterial transfer [7]. In our study, this was true in the univariate analyses, as the annual average temperature of a country increased, so too did the level of blood culture QREC (%). However, it lost its significance in the multivariable models. It is also possible that average temperature is a proxy for other socioeconomic factors. Although not the direct causal mechanism, higher temperatures have previously been associated with lower levels of income and reduced quality of water and sanitation [30, 31]. One of the ongoing concerns of climate change is the risk that increased temperatures will have on vector-borne diseases. As temperatures rise, so too will the incidence of vector-borne diseases, thus resulting in increased use of antibiotics, and thus, potentially, an increase in AMR [32].

Unlike other studies, we included livestock and crop production index, which reflect the growth in agricultural production relative to a baseline period. We found them to be positively associated with blood culture QREC (%) in univariate and multivariable analyses of all countries combined but showed no significance when analysed in the high and middle-income models only. Often policy makers focus only on the misuse of antimicrobials amongst the human population, but antimicrobials are also used extensively in the agricultural, farming and aquaculture industry. As demonstrated above, as the quantity of livestock and agricultural production increases in a country, there is an associated increase in blood culture QREC (%). This could be as a result of increased use of antimicrobials in these industries and subsequent run-off into the environment.

### Strengths and limitations

The major downfall of our study was the quantity of data available and the non-randomness of some of the missing data. The data we used was also cross-sectional which prevented us from capturing relationships which take place with a time lag. Data collection is a costly process and one that requires a stable economy and political state. Consequently, developed countries are over-represented, and indeed, there was only one low-income country with data on blood culture QREC (%). While we can draw relatively certain conclusions on high-income countries, it is possible that there are different drivers in low-income countries for which we have little data, and even within the middle-income group for which we probably had insufficient data. The collection of national data on AMR via the WHO GLASS project is a relatively new endeavour and one that requires large input from governments and health departments. Several countries have enrolled in GLASS, but it will take many years before it becomes routine data collection internationally.

Our study only looked at blood culture QREC (%) as a marker of AMR. It has been shown that pathogens have different responses to antimicrobial use and it is possible that by focusing on a single drug-pathogen combination that the results may have limited generalisability [33]. It has also been demonstrated that intense and repeated use of antibiotics has a stronger association with AMR that extensive low-intensity use [34]. It was out of the scope of this study to include the distribution and intensity of antimicrobial use, but we are aware that this may be another potential source of bias. Bacteria also display either disjoint or concurrent resistance and if we had looked at other drug-pathogen combinations, we may have found that the effect of a variable on one drug-pathogen combination might have differed to the effect on another combination and, indeed, the overall levels of AMR.

In addition, while we included diverse variables, it is possible that some statistical errors of endogeneity exist. There is potential for reverse causality where factors associated with AMR also influence prescribing practices or where the level of AMR influences the independent variables. It is also possible that some factors that are drivers for AMR were omitted or unobserved or that some of the included variables exhibit simultaneity. For example, it is possible that countries with higher income have both less corruption, greater access to antimicrobials and improved sanitation and water infrastructure. Indeed, it is unlikely that corruption itself causes higher levels of AMR, this association likely reflects a range of socioeconomic factors which are correlated with perceived corruption. We tried to account for potential multi-collinearity by calculating the Variance Inflation Factor (< 6) which demonstrated the likelihood of the analysed variables being correlated with each other. When performing multivariate analysis, there is always a balance between potentially omitting relevant drivers or including too many, resulting in simultaneity or reversed causality, and we acknowledge this as a limitation.

This aside, our study analysed a large number of socioeconomic and environmental factors associated with AMR. Our study is the first we know to perform multivariable analyses where we stratified drivers of AMR by different levels of income. Although there is uncertainty about details in our results, they did indicate there might be different main drivers of AMR in different economic settings, pointing to the need for differentiated policies to fight AMR. There has been a call for environmental regulation and monitoring to be included in AMR action plans, although a lack of understanding of the drivers and pathways of AMR and the differences between these drivers in different settings has resulted in an inability to include many of them in policy until now [35]. As other research has demonstrated, AMR action plans also need to bridge the worlds of antibiotic use in health care, agriculture and aquaculture [36]. Our research emphasises that there cannot be a single action plan that meets the needs of all countries globally and that AMR action plans need to take into account different socioeconomic, geospatial and environmental factors while acknowledging gaps in the surveillance capacity of lower economic settings.

#### Future research

We have identified gaps in the research, namely, in the amount of data available on AMR in low-income countries. We recommend steps to improve AMR surveillance in low-income countries. If such data becomes available, we suggest running further multivariable analyses of AMR drivers in low-income countries in order to guide political prioritization of action against AMR.

## Conclusion

AMR is a global health concern. Using blood culture QREC (%) as a marker of AMR in a country, we found that corruption and antibiotic use were strong predictive factors for both high- and middle-income countries, with sanitation services also playing a role in middle-income countries. The association between corruption and AMR likely reflects a range of socioeconomic factors which are correlated with the variables and the mechanism behind these associations needs to be explored in more detail. Especially for low-income countries, it is imperative to improve AMR surveillance, in order to guide political prioritization of action against AMR.

## Supplementary Information

**Additional file 1.**

## Data Availability

The datasets used and analysed during the current study are available from the corresponding author on reasonable request.

## Abbreviations

AMR: Antimicrobial Resistance
CDDEP: Centre for Disease Dynamics, Economics and Policy
CLSI: Clinical Laboratory Standards Institute
DDD: Defined Daily Doses
ESBL: Extended-spectrum beta-lactamase
EUCAST: European Committee on Antimicrobial Susceptibility Testing
GLASS: Global Antimicrobial Resistance Surveillance System
GNI: Gross National Income
IQR: Interquartile ranges
JMP: Joint Monitoring Programme
MCAR: Missing Completely at Random
Mg/PCU: Mg per population correction unit
OIE: World Organisation for Animal Health
QREC (%): Proportion of Quinolone-resistance *Escherichia coli* in blood culture samples
UNICEF: United Nations Children’s Fund
VIF: Variance Influence Factor
WASH: Water Supply, Sanitation and Hygiene
WHO: World Health Organisation
