# Correction to: A multivariable analysis of the contribution of socioeconomic and environmental factors to blood culture *Escherichia Coli* resistant to fluoroquinolones in high- and middle-income countries

**DOI:** 10.1186/s12889-022-12896-5

**Published:** 2022-03-18

**Authors:** Amy Booth, Astrid Louise Wester

**Affiliations:** 1 Faculty of Health Sciences, University of Cape Town, Rondebosch, Cape Town, 7700 South Africa; 2 Centre for Antimicrobial Resistance Research, and Division of Infection Control, Norwegian Institute of Public Health, Lovisenberggata 8, 0456 Oslo, Norway

**Correction to: BMC Public Health 22, 354 (2022)**

**https://doi.org/10.1186/s12889-022-12776-y**

The original publication of this article contained an incorrect Fig. 1. The incorrect and correct Fig. 1 are included in this correction article (see Fig. 1 and Fig. 2).

The original article has been updated.

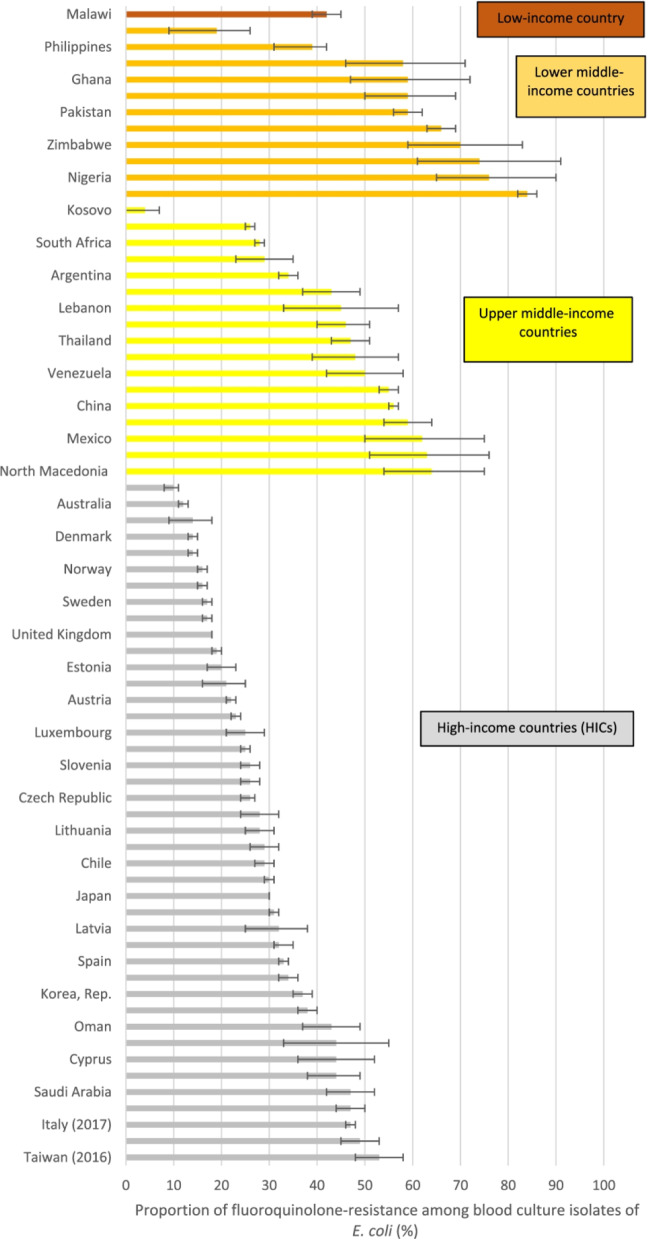



Fig. 1 Incorrect version of figure 1 as originally published

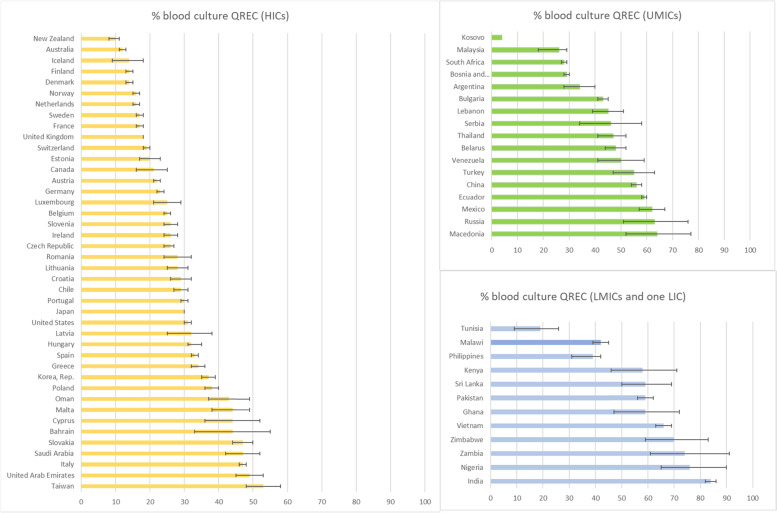



Fig. 2 Correct version of figure 1 as corrected

