# Supplementary material for: A multivariable analysis of the contribution of socioeconomic and environmental factors to blood culture Escherichia coli resistant to fluoroquinolones in high- and middle-income countries
Source: BMC Public Health. 2022 Feb 19;22:354. doi: 10.1186/s12889-022-12776-y (PMC8857829; doi:10.1186/s12889-022-12776-y)
Supplement: Supplementary file 1 — Additional file 1. [file 12889_2022_12776_MOESM1_ESM.docx]

**Appendix:**

| **Supplemental Table 1: Univariate linear regression of independent variables for blood culture QREC (%) isolates tested in all, high-income and middle-income countries** | | | |
| --- | --- | --- | --- |
| **Variable (Units)** | **Regression Coefficient** | | |
|  | **All Countries** | **High Income Countries** | **Middle Income Countries §** |
| **Unsafely Managed Sanitation (%)** | 0·49 *** | 0·07 | 0·26 ** |
| **Unsafely Managed Water (%)** | 1·0 *** | 2·07 | 0·44 * |
| **Human Fluoroquinolone Consumption (DDD/ 1000 population/ year)** | 0·02 *** | 0·01 *** | 0·01 |
| **Total Human Antimicrobial Consumption (DDD/ 1000 population/ year)** | -0·00 | 0·00 * | -0·00 |
| **Animal Antimicrobial Consumption (mg/PCU)** | 0·08 * | 0·09 ** | 0·01 |
| **Population Density (population per square kilometre)** | 0·01 | 0·01 ** | 0·02 |
| **Gross National Income (US dollars)** | -0·00 *** | -0·00 *** | -0·00 |
| **Corruption Perceptions Index (score 1-100)** | -0·68 *** | -0·61 *** | -0·88 ** |
| **Education Level (years)** | -5·4 *** | -4·78 *** | -2·53 * |
| **Healthcare Access and Quality (score 1-100)** | -0·89 *** | -1·27 *** | -0·52 ** |
| **Average Annual Temperature (degrees Celsius)** | 1·17 *** | 0·96 *** | 0·31 |
| **Livestock Production Index** | 0·18 *** | 0·08 | 0·03 |
| **Crop Production Index** | 0·19 ** | -0·03 | 0·27 * |
| **Aquaculture Production Index (metric tons)** | 0·00 | -0·00 | 0·00 |

***** p<0.01, ** p<0.05, * p<0.1**

§ Malawi included
